# Global Genetics and Invasion History of the Potato Powdery Scab Pathogen, *Spongospora subterranea* f.sp. *subterranea*

**DOI:** 10.1371/journal.pone.0067944

**Published:** 2013-06-28

**Authors:** Rebecca D. Gau, Ueli Merz, Richard E. Falloon, Patrick C. Brunner

**Affiliations:** 1 Plant Pathology, ETH Zurich, Zurich, Switzerland; 2 New Zealand Institute for Plant & Food Research Limited, Christchurch, New Zealand; 3 Bio-Protection Research Centre, Lincoln University, Lincoln, New Zealand; North Carolina State University, United States of America

## Abstract

*Spongospora subterranea* f. sp. *subterranea* (*Sss*) causes two diseases on potato (*Solanum tuberosum*), lesions on tubers and galls on roots, which are economically important worldwide. Knowledge of global genetic diversity and population structure of pathogens is essential for disease management including resistance breeding. A combination of microsatellite and DNA sequence data was used to investigate the structure and invasion history of *Sss*. South American populations (four countries, 132 samples) were consistently more diverse than those from all other regions (15 countries, 566 samples), in agreement with the hypothesis that *Sss* originated in South America where potato was domesticated. A substantial genetic differenciation was found between root and tuber-derived samples from South America. Estimates of past and recent gene flow suggested that *Sss* was probably introduced from South America into Europe. Subsequently, Europe is likely to have been the recent source of migrants of the pathogen, acting as a “bridgehead” for further global dissemination. Quarantine measures must continue to be focussed on maintaining low global genetic diversity and avoiding exchange of genetic material between the native and introduced regions. Nevertheless, the current low global genetic diversity of *Sss* allows potato breeders to select for resistance, which is likely to be durable.

## Introduction

There have been many deliberate or accidental anthropogenic introductions of organisms beyond their original geographical ranges [1]. Many important crops have been intentionally taken from their regions of origin and introduced to other suitable environments around the world, because of their value in food and fiber production [2]–[4]. These introductions have often been accompanied by unintentional introduction of plant pathogens. Furthermore, increasing global trade in plant products carries the danger of introduction of pathogens, providing recurrent opportunities for new invasions [5]–[7]. These pathogens have the potential to cause severe economic losses to crops, ornamental plants, or forests, and can lead to severe problems in human or livestock nutrition [8]–[9].

There are numerous examples of invasive plant pathogens that have been introduced and distributed into new areas beyond their native ranges through human activities. *Puccinia striiformis* f. sp. *tritici*, the causal agent of stripe rust of wheat, was introduced into the USA almost 100 years ago and caused severe economic losses [10]. The introduction of *Phytophthora ramorum* was also human-mediated, leading to sudden oak death in North America [11]. The most infamous example is *Phytophthora infestans,* the causal agent of potato late blight. This disease was first reported in the USA 1843, and soon after appeared in Ireland where it led to the Irish potato famine with well-recognised and documented consequences of mass human starvation and forced migration [12]. Once introduced in Europe, *P. infestans* was distributed worldwide via the international seed potato trade.

Invasive plant pathogens often successfully establish in new regions and spread over large areas [13]. Despite the importance of these invasions, relatively little is known about the modes, timing or frequency with which they have occurred. Knowledge of these factors is important for prediction, prevention, and response to additional introductions [9]; [14]. Molecular genetic data can be useful for the evaluation of the reasons for invasion success. It is an adjunct to ecological knowledge helping to elucidate sources and routes for invasions and to identify the patterns of dispersal and the genetic composition of founding populations.It has long been accepted that populations of invasive alien species have reduced genetic variation compared to their source populations [15], and founder effects occur due to small population size in the introduction and establishment phases of biological invasions. This should hamper successful invasions, due to limited ability to respond to selective pressures. Usually, lag phases are expected between colonization and expansion, and during these lags multiple further introductions are thought to be needed to allow evolution of new adaptations [15]. However, there are several examples of successful invasive plant pathogens with low genetic variability, including the potato pathogens *P. infestans*, and the wilt-causing bacteria *Burkholderia solanacearum* and *Ralstonia solanacearum* [16]–[17]. A concept from invasion theory states that some invasive species are able to establish in a new territory after only one or a few introduction events and are then cut off from the source population. Further spread is exclusively outgoing from this introduced population to other regions. This phenomenon - called the “bridgehead effect” by Lombaert *et al*. [18] - suggests that genetic diversity is not essential for invasion success, and that rapid adaptive evolution is possible despite strong bottlenecks and single introduction events. Although such invasive pathogens may each have a clonal genetic structure, different phenotypes can be observed under varying climatic or environmental conditions, as observed by Fry and Goodwin [19] for *P*. *infestans*. Therefore, it is important to thoroughly characterize even clonal invasive plant pathogen species.

*Spongospora subterranea* f. sp. *subterranea* (Cercozoa, Plasmodiophoridae*;* hereafter abbreviated as “*Sss”*), is the causal agent of powdery scab, an economically important disease complex of potato and the vector of Potato mop-top virus (PMTV), the cause of "spraing" in potato tubers. Powdery scab usually refers to the scabby lesions caused by the pathogen on potato tuber surfaces, but also galls on potato roots are a symptom of infection of these organs by *Sss*. The main host species of *Sss* is potato, *Solanum tuberosum* ssp. *tuberosum*. Other important hosts are *Solanum tuberosum* ssp. *andigena* and *Solanum phureja*, both of which are potatoes cropped in South America, the native region of potato, in the higher altitude areas of the Andes. Alternative hosts are wild potato species (South America) and other solanaceous plants, e.g. nightshade (*Solanum nigrum*), a common weed in potato production [20]. Different potato cultivars show differences in susceptibility to root and tuber infection [21]. In Colombia it is well recognized that root galls are commonly formed on *Sss*-infected potato plants, and that tuber lesions caused by *Sss* are less frequently observed [22]. In most of the regions, where potato was introduced, both symptoms occur. The mechanisms behind the differences in susceptibility to the two forms of disease caused by *Sss* remain to be elucidated.

**Figure 1:**
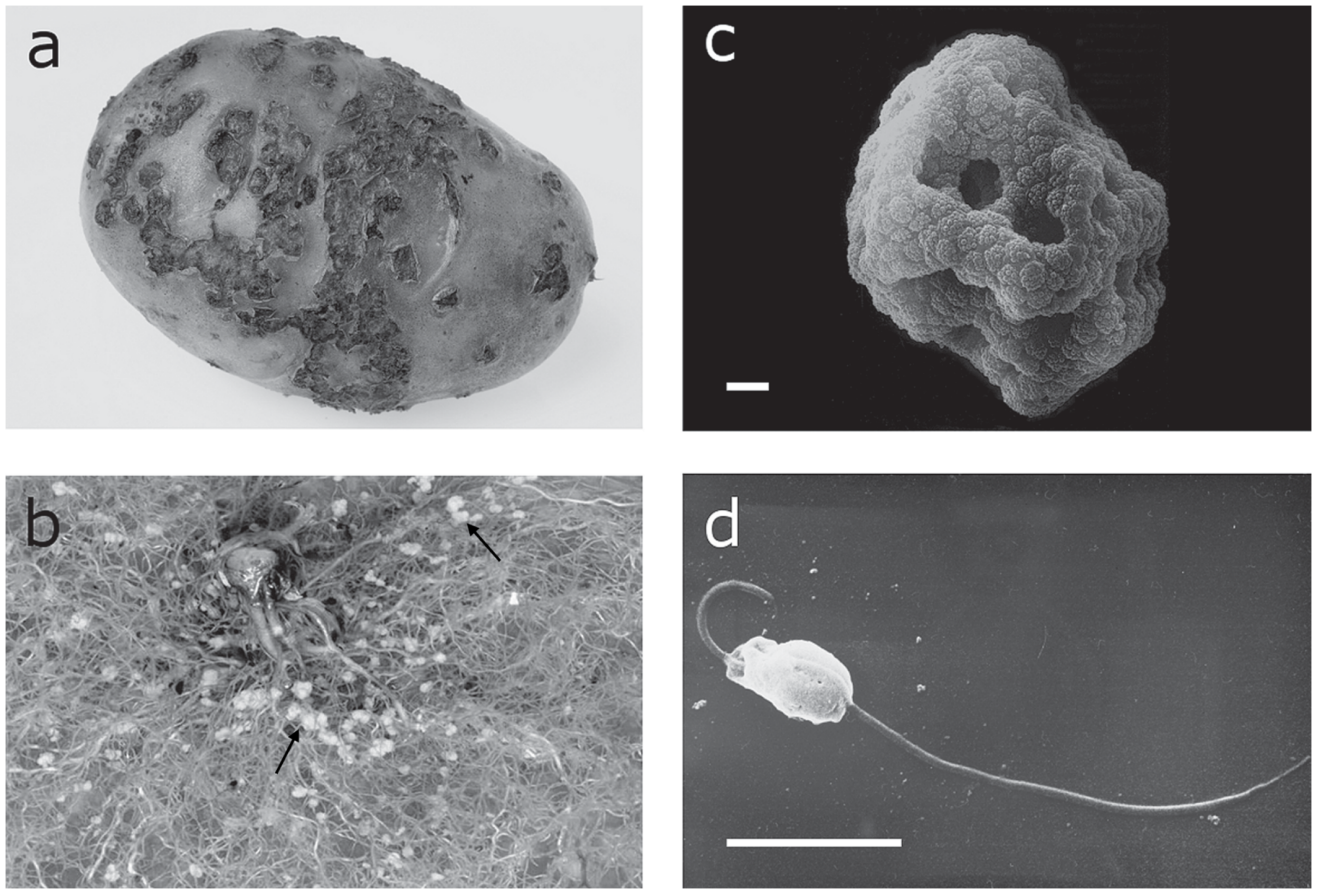
Disease symptoms caused by, and life cycle stages of, *Spongospora subterrane* f.sp. *subterranea*. Disease symptoms on potato: a) powdery scab lesions. b) root galls. and life cycle stages of the pathogen: c) Sporosorus containing resting spores. d) Single, biflagellate primary zoospore. Bars = 5 µm. Pictures a) and b) taken by R. Lamberts.

The life cycle of this obligate soilborne biotroph prevents natural long distance dispersal, because resting spores, aggregated in sporosori, are formed in the soil in lesions on the surface of potato tubers and in galls on roots (Figs. 1a–c). Furthermore, the biflagellate primary zoospores (Fig. 1d), which emerge from resting spores, as well as the secondary zoospores produced in zoosporangia in host root cells, are able to swim only short distances in moist soil to infect new tissue. This reduced dispersal ability should lead to genetically distinct populations due to the lack of homogenizing gene flow. However, there is considerable global trade in potatoes, and movement of *Spongospora*-infected seed potatoes is therefore likely to be responsible for successful short and long distance dispersal of the pathogen [23].

Powdery scab is difficult to manage because contaminated soils remain infectious for many years due to the formation of numerous, highly resistant resting spores. From about the 1950s to the 1980s, seed potato tubers were routinely treated with mercury-containing pesticides to effectively protect potato crops from the disease [24]. These treatments were suspended for human health and environmental reasons, and have not been replaced by fully effective seed tuber treatments. Breeding of resistant potato cultivars will play an important role in controlling the disease [23]. Until now, plant breeders screening cultivars and lines for susceptibility to powdery scab have been doing so without knowledge of genetic variability in *Sss*, and little is known about the role of sexual recombination in its lifecycle. The assumption that sporosori are the product of sexual recombination [25] remains to be demonstrated. Very few studies have addressed genetic variation of *Sss*, mainly for detection and diagnostic purposes, using variation in the internal transcribed spacer (*ITS*) region [26]–[27]. Because *ITS* is a rather conserved marker, these studies detected only slight genetic diversity among a limited number of samples. Only one reported study has used randomly amplified polymorphic DNA (RAPD) analysis to evaluate restriction fragment length polymorphism (RFLP) data [28], but only for North American samples from eight sites with each site represented by three samples. Variation between, but not within, geographic locations has been detected. These studies have provided little insight into the present genetic population structure of *Sss* on a global scale.

One goal of the present study was to provide the first broad scale population genetic study of an important plasmodiophorid pathogen, based on newly-developed microsatellite markers and sequences of the *actin* gene and *ITS* region. Data were obtained from sporosorus samples originating from six continents and many potato producing regions. It has been suggested that South America is the native region of origin of *Sss* [29]. Our second goal was to test the hypotheses that the pathogen was introduced from South America to Europe, and was subsequently dispersed with Europe acting as a bridgehead, through colonial [30] and/or contemporary seed potato trade [31] to the other introduced regions.

These new insights will expand knowledge of the pathogen and are required to improve powdery scab management, particularly for designing and implementing suitable quarantine strategies and for developing plant resistance as a sustainable method for practical powdery scab management.

## Materials and Methods

### Samples and DNA Extraction

Collecting of populations of *Sss* followed an hierarchical sampling scheme [32]. The 19 countries of sample origin, combined into six regions and sample sizes per region are listed in Table 1 and in more detail in Table S1. To simplify further naming in this paper, South American samples are specified as ‘native’ and all others samples as ‘introduced’. Total DNA from dried lesion scrapings from potato tubers or potato root galls was extracted using the cetrimonium bromide (CTAB) method [33] or the QIAgen DNeasy Plant mini kit. Prior to further analyses, the presence of *Sss* DNA in the extraction was confirmed using a *Sss*-diagnostic PCR that amplifies a fragment of the *ITS* region [26].

**Table 1:** Number of *Spongospora subterranea* f.sp. *subterranea* samples collected, genotyped and sequenced, sorted by geographical region.

Region	Specified as	Countries	No. of samples	No. of genotyped samples	No. of sequenced samples
Europe	Introduced	Switzerland, Germany, Netherlands, Norway, Sweden, Scotland, Iceland	215 (8)^a^	215	69
Africa		South Africa	57 (0)	57	12
Asia		South Korea, Japan, Pakistan, Sri Lanka	98 (6)	98	38
Australasia		Australia, New Zealand	170 (3)	170	40
North America		United States	26 (0)	26	11
South America	Native	Colombia, Venezuela, Peru, Ecuador	93 (39)	127	132
Total			659 (56)	693	302

a Number of root gall samples in brackets.

### Nucleotide Sequences and Phylogenetic Reconstruction

The taxonomy and species diversity of the *Plasmodiophoridae* is poorly investigated. Since we compared samples from a broad range, including different host plants, tuber lesions and root galls, we first conducted a phylogenetic analysis to avoid the comparison of cryptic species. A subset of 302 *Sss* samples representing all regions was analyzed for nucleotide sequence variation at the *ITS* region (389 bp) and the partial *actin* gene (615 bp). A standard PCR protocol was used to amplify *ITS* using the primer pairs Spo8 and Spo9 [26]. A nested PCR using newly designed “outer” and “inner” primer pairs based on the published *actin* sequence (AY452193.1) had to be applied to amplify the *actin* gene (Table S2). Products were sequenced with an ABI 3730 xl sequencer (Applied Biosystems). Sequences were edited using SEQUENCHER (Gene Codes Corporation). BLASTN searches [34] were carried out against the GenBank data base to verify that the sequences were not from other organisms. The sequences of the two genes were concatenated and aligned using ClustalW [35]. ITS and/or *actin* sequences of other Plasmodiophorids were retrieved from GenBank as outgroups, including *Spongospora subterranea* f. sp. *nasturtii*, the closest known relative of *Sss*. ITS and actin haplotypes generated in this study are deposited in GenBank under accession numbers KF018341–KF018378.

Maximum Likelihood (ML) phylogenetic trees were constructed using MEGA version 5 [36]. The ML analyses were performed using the Kimura-2-parameter model with a discrete Gamma with five rate categories. The model search algorithm implemented in MEGA selected this model as having the lowest Bayesian Information Criterion (BIC) score, which is considered to best describe the nucleotide substitution pattern. All positions with less than 70% site coverage were eliminated. Statistical node support was estimated using 500 bootstrap replications. We also constructed a parsimony-based haplotype network using TCS [37] and the haploNet function of R (http://www.r-project.org/), to improve visualization of the haplotype relationships and frequencies across regions.

### Microsatellite Library Construction

An enriched microsatellite library [38] was established to develop microsatellite primers for *Sss*. In short, *Sss* DNA from a Swiss tuber lesion sample was digested with *Rsa*I and *Xmn*I to obtain ca. 500 bp blunt-ended fragments. From these fragments an enriched microsatellite library was produced using magnetic beads (MyOne T1 Streptavidin Dynabeads, Invitrogen) and biotinylated oligonucleotides representing the microsatellite motives (AT)_10_, (CT)_10_, (TTG)_8_ and (TCG)_8_. Ligation and cloning of enriched fragments were performed, using the TA Cloning® Kit (Invitrogen). Colonies containing plasmids with an insert (white color) were sequenced using an ABI 3730 xl sequencer (Applied Biosystems) and screened for microsatellites. A BLASTN search [34] with all obtained sequences was performed against the GenBank database to identify non-specific *Sss* fragments (e.g. from potato or soil organisms). The web-based PRIMER3 program [39] was used to design the specific microsatellite primers.

PCR amplifications using the msat246 primers produced two fragments of 140 bp and 160 bp respectively. Cloning and re-sequencing suggested a duplication of this locus in the genome with the shorter fragment having a 20 bp deletion. Since the deletion was located outside of the microsatellite motif (CAA) and the two loci were unlinked, both were scored and analyzed.

### Microsatellite Analyses and Population Structure

Six polymorphic microsatellite loci yielding unambiguous PCR products were selected to genotype 693 *Sss* samples. Separate PCRs were carried out for each locus using fluorescent-labeled primers (Table S2). Amplicons were separated using either an ABI 3730 xl or an ABI 3130 sequencer (Applied Biosystems). Data processing and calling of allele-sizes was performed using internal GeneScan LIZ600 standards and the GENEMAPPER software (both Applied Biosystems).

Based on observation of either one (homozygote) or two microsatellite alleles (heterozygote) among the samples, we assumed that *Sss* is a diploid organism, and all analyses were performed accordingly. Isolates with identical multilocus genotypes (MLG; i.e. possessing the same allele at all microsatellite loci) were considered distinct clones and only one MLG was retained per population for subsequent analyses. We used the program GENODIVE [40] assuming an infinite allele model of microsatellite evolution to calculate allele frequencies (provided in Table S3), site- specific genotypes, the clonal fraction which describes the proportion of individual samples originating from asexual reproduction, Nei’s genotypic diversity [41] and gene diversity for each region. Estimates were corrected for differences in sample size using the rarefaction [42]. Significance of differences between regions was assessed using the implemented bootstrapping approach. Linkage equilibrium of MLGs (i.e. the random association of microsatellite loci) was assessed as a proxy to estimate the amount of sexual recombination by estimating the index of association I_A_^S^ using LIAN 3.5 [43].

The population structure of *Sss* was explored on the individual and the region levels using the multivariate clustering approach of principle component analysis (PCA) based on the covariance matrix of allele frequencies. The optimal number of clusters at the individual level was further assessed using K-Means clustering [44] as implemented in GENODIVE. The method uses a pairwise matrix of distances between all observations and divides these observations into an *a priori* assigned number (k) of groups in such a way that the among-groups Sum of Squares is maximized. We used the option of simulated annealing based on a Monte Carlo Markov Chain (MCMC; one million steps) and repeated the analysis five times to ensure that the clustering did not get stuck in local optima. The optimal value of k was inferred from the Calinski & Harabasz [45] pseudo-*F*-statistic, which is particularly suited when there is non-random mating, and for clustering individuals [46]. Differentiation on the region level was estimated as pairwise *F_ST_* values using the method of Weir and Cockerham [47], and the significance of genotypic differentiation was assessed using the permutation approach (10,000 iterations) implemented in GENODIVE.

### Inferring Migration History

We inferred migration rates between regions on two temporal scales. Past (long-term) migration rates were estimated using the maximum-likelihood approach implemented in MIGRATE [48]. Based on the *F_ST_* analyses that suggested non-significant genetic differentiation (see results below), Colombia tuber lesions and Venezuela tuber lesions were pooled into “South America tuber lesions”. The starting values for the migration rates were estimated based on pairwise *F_ST_*. Markov chain settings were ten short chains, three long chains with a burn-in of 10,000 trees and averaging over long chains. We applied a three-temperature heating scheme and selected the Brownian mutation model for microsatellite evolution. Convergence of parameter estimates was controlled by checking the MCMC process for high acceptance ratios (>95%), for stationarity of data-likelihood estimates, and by running the entire analyses three times to ensure consistency of results. However, most of the pairwise comparisons yielded inconsistent results, e.g. while in one run region A was inferred as the sink and region B as the major source of gene flow, repeating the same analysis resulted in the opposite outcome. This is likely to be due to the shallow population structure among the introduced regions. We therefore restricted our analyses of historic migration rates to test the hypothesis of a historic introduction of *Sss* from South America to Europe through colonial trade [29]; [30], and in a second analysis between South America and the pooled introduced regions. Both comparisons resulted in consistent results between repetitions.

Estimates of recent migration rates were performed using the software BayesAss v. 3 [49]. The Bayesian approach estimates the proportion of genotypes in a population composed of migrants over the last few generations. After checking preliminary runs that log-probability fluctuations were restricted to the burn-in phase, indicating good convergence, the final mixing parameter for allele frequencies was set to 0.3 and the mixing parameter for inbreeding coefficients was set to 0.2. We allowed for a burn-in of 1,000,000 iterations and a MCMC sampling of 10,000,000 iterations. The hypothesis to be tested was that Europe acted as a bridgehead for subsequent dispersal of *Sss* to the other introduced regions, for example through contemporary seed potato trade [31]. All pairwise comparisons using this approach yielded consistent results.

## Results

### Phylogeny

We used the concatenated nucleotide data from the *actin* and ITS region to reconstruct the phylogenetic relationship of *Sss* and other Plasmodiophorids. The distribution of distinct haplotypes was extremely skewed. Of the 19 distinct concatenated haplotypes (cHap), 17 were found in the native regions and two in the introduced regions. Of these two, cHap6 was found in all introduced regions and accounted for 96% of haplotypes found in these regions. None of the cHaps were shared between introduced and native regions. A detailed list of haplotype distribution is given in Table S4 and visualized in Figure S1.

**Figure 2:**
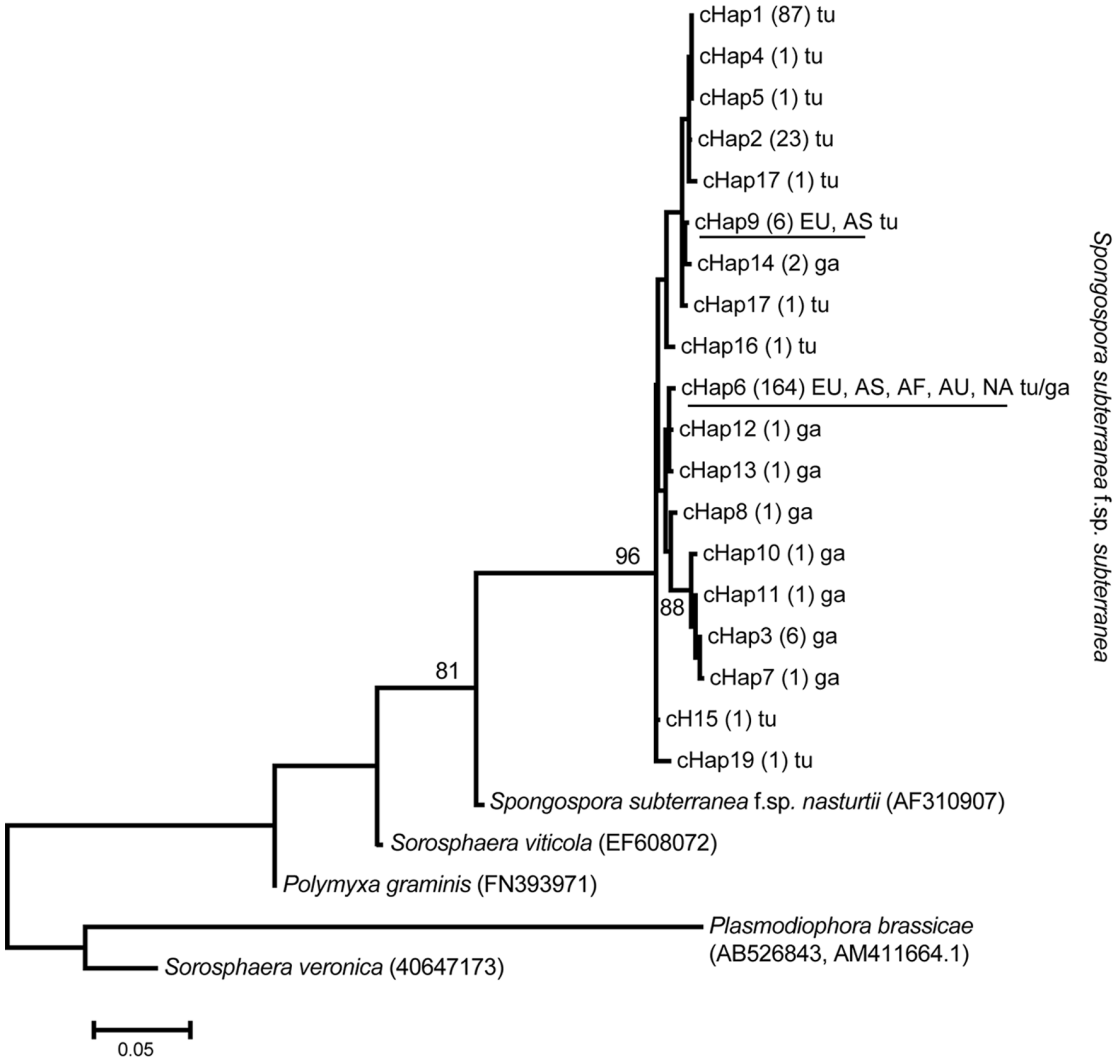
Inferred phylogeny of *Spongospora subterranea* f.sp. *subterranea* and other Plasmodiophorids. The evolutionary relationship of *Spongospora subterranea* f.sp. *subterranea* and other Plasmodiophorids was inferred using the Maximum Likelihood method on the concatenated ITS and *actin* sequences. The bootstrap consensus tree inferred from 500 replicates is taken to represent the evolutionary history of the taxa analyzed. Branches corresponding to partitions reproduced in less than 50% bootstrap replicates are collapsed. The tree is drawn to scale, with branch lengths measured in the number of substitutions per site. The number of samples possessing a particular haplotype is given in parenthesis. The underlined haplotypes were detected in the introduced regions Europe (EU), Africa (AF), Asia (AS), Australasia (AU), and North America (NA). tu, haplotypes detected from tuber lesions; ga, haplotypes detected from root galls. GeneBank accession numbers are given for the outgroup species.

The reconstructed ML tree (Fig. 2) clustered all *Sss* haplotypes in a well supported monophyletic clade (96% bootstrap replicates) and clearly distinct from its close relative species *S. subterranea* f.sp. *nasturtii*. Average pairwise distances among cHaps were low (0.017 substitutions/site) and there was no significant substructure within the *Sss* clade. However, there was a tendency of cHaps to cluster according to inoculum as most haplotypes collected from tuber lesions (tu) formed one group, and most haplotypes collected from root galls (ga) clustered in a second group. CHaps are inoculum-specific in the native region, i.e. there were no shared haplotypes between root galls and tuber lesions. In contrast, cHap6 that accounted for most haplotypes in all introduced regions was associated both with root galls and tuber lesions.

### Genetic Diversity

A complete list of microsatellite allele frequencies for the six regions is available in Table S3. Measures for the genic diversity are summarized in Table 2. All results of genic diversity showed significantly lower values for the introduced regions compared to the native regions. The six microsatellite loci had two to 12 alleles per locus (average of six alleles per locus) and 35 alleles in total. A total of 131 different multilocus genotypes (MLG) were detected among the 693 samples analyzed in the six regions. South America possessed most of the detected genotypes. Out of 127 genotyped samples from this region, 82 MLGs were detected, resulting in the smallest clonal fraction (Table 2). Of these 82 distinct MLGs, 81 were site-specific, found only in South America. Within South America, the root gall (cf = 0.226) and tuber lesion samples (cf = 0.382) had similar diversities. In contrast, all introduced regions had significantly greater clonal fractions, ranging from 0.91 (Australasia) to 0.63 (Africa). No distinct MLGs were detected for gall-derived samples among the introduced regions. Given this finding and the small sample sizes (Table 1), we did not conduct separate analyses for gall-derived samples for the introduced regions. Most striking was the low number of site-specific genotypes ranging from one to 13 among the introduced regions compared to 81 detected in South America. Only one multilocus genotype was shared between introduced regions and the native region. In the 566 samples of the pooled introduced regions, 49 MLGs were detected, resulting in a significantly greater clonal fraction (cf = 0.913) compared to South America (Table 2). No significant differences in pairwise comparisons between the introduced regions were found, with exception of North America. North America had a genetic diversity of G = 0.151, which is approximately five to six times less than found in any other region.

**Table 2:** Genetic diversity parameters of *Spongospora subterranea* f.sp. *subterranea* within the sampled regions, determined using six microsatellite markers.

Region	N^a^	cnum^b^	ssg^c^	cf^d^	G^e^	H^f^	I_A_ g
Europe	215	26	11	0.88	0.885	0.225	0.0339***
Africa	57	21	13	0.63	0.917	0.234	0.0144^ns^
Asia	98	16	7	0.84	0.835	0.249	0.0673***
Australasia	170	16	2	0.91	0.878	0.21	0.0131***
North America	26	3	1	0.89	0.151	0.013	na
Total Introduced	566	49	34	0.91	0.91	0.235	0.0205**
South America Root galls	39	29	26	0.23	0.983	0.391	0.0630**
South America Tuber lesions	88	55	54	0.38	0.972	0.314	0.0269*
Total Native	127	82	81	0.35	0.985	0.461	0.1138***

a N = Sample size.

b cnum = Number of multilocus genotypes.

c ssg = Site-specific genotypes; clones specific to a region and not shared with other regions.

d cf = Clonal fraction; proportion of individual samples originating from asexual reproduction.

e G = Nei’s corrected diversity (genotypic diversity).

f H = Nei’s Gene Diversity.

g I_A_ = Index of association to tests the null hypothesis of linkage equilibrium for multilocus data. Significance of deviation from equilibrium expectations are indicated by asterisks. *, p<0.05, **, p<0.01, ***, p<0.001; ns = non-significant; na = not enough diversity for estimation.

The standard index of association (I_A_^S^) for each regions and pooled regions was measured to test for statistical independence amongst alleles at each of the six microsatellite loci (Table 2). Based on this method, only the I_A_^S^ = 0.0144 for Africa did not significantly deviate from linkage equilibrium. In contrast, I_A_^S^ estimates for all other regions indicated significant linkage disequilibrium, suggesting that *Sss* is only very rarely undergoing sexual recombination.

### Population Structure

**Figure 3:**
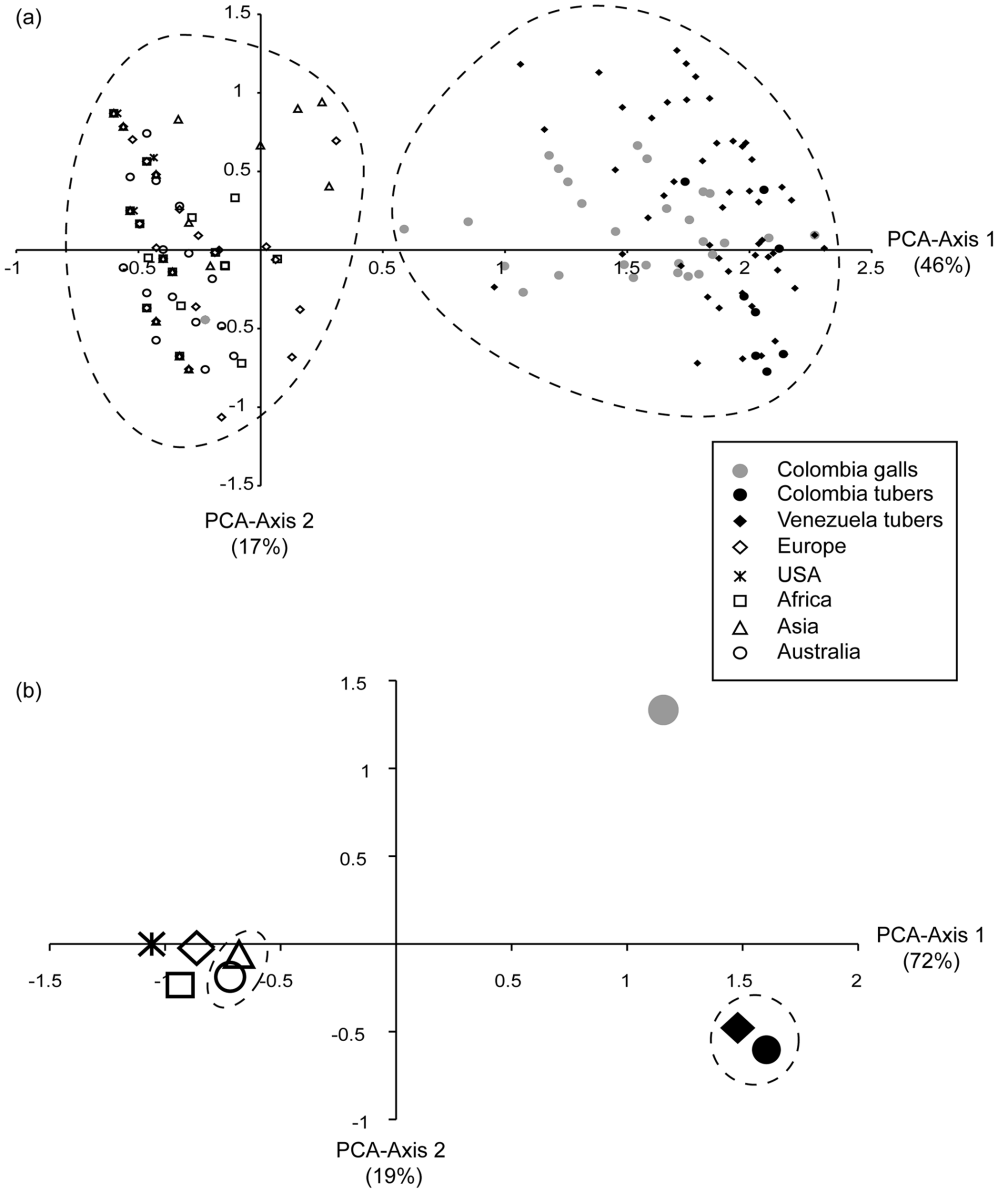
Principle Component Analyses (PCA) of *Spongospora subterranea* f.sp. *subterranea* multilocus genotypes. The analyses based on covariances of allele frequencies. (a) PCA performed on individuals resulted in axis 1 explaining 46% and axis 2 explaining 17% of the genetic variation. The dashed lines encircle the two clusters identified by K-means clustering. (b) PCA performed on regions resulted in axis 1 explaining 72% and axis 2 explaining 19% of the genetic variation. The dashed lines encircle regions that are not differentiated based on *F_ST_* analysis.

The microsatellite data was subjected to PCA on two levels to explore the population structure of *Sss* (Fig. 3). On the individual level, the first two PCA axes, respectively, explained 46% and 17% of the total genetic variation. While no sub-clustering by region was observed, the canonical plot showed clear distinction between individuals from the introduced regions and individuals from the native region. These two global clusters were confirmed as the optimal number of groups using the K-means approach on the individual level (Fig. 3a).

On the regional level, PCA-Axis 1 (72%) and Axis 2 (19%) together explained 91% of the total genetic variation. The clustering confirmed the shallow sub-structure among the introduced regions, grouping the five regions very closely together. However, based on the pairwise *F_ST_* estimates (Table 3) only Australasia and Asia were not significantly differentiated from each other. Also in contrast to the individual level analyses, PCA and *F_ST_* analyses detected significant substructure among the native South American regions. Here, independent of geographic proximity, the tuber population from Colombia and the tuber population from Venezuela formed a cluster distinct from the gall population from Colombia (Fig. 3b). In contrast, we found no evidence of genetic differences in *Sss* related to different hosts in both neither the sequence-based phylogeny reconstruction and the microsatellite based cluster analysies.

**Table 3:** Pairwise estimates of *F_ST_* between sampled regions (above diagonal) and corresponding p-values (below diagonal).

Region	EU	AF	AS	AU	NA	CO lesions	CO galls	VE lesions
Europe	–	0.073	0.081	0.116	0.176	0.710	0.654	0.645
Africa	<0.001	–	0.043	0.041	0.303	0.689	0.617	0.607
Asia	<0.001	<0.001	–	0.006	0.361	0.667	0.612	0.609
Australasia	<0.001	<0.001	0.028^ns^	–	0.394	0.704	0.663	0.652
North America	<0.001	<0.001	<0.001	<0.001	–	0.877	0.712	0.691
Colombia tuber lesions	<0.001	<0.001	<0.001	<0.001	<0.001	–	0.507	0.071
Colombia root galls	<0.001	<0.001	<0.001	<0.001	<0.001	<0.001	–	0.448
Venezuela tuber lesions	<0.001	<0.001	<0.001	<0.001	<0.001	0.002^ns^	<0.001	–

ns non-significant after Bonferroni correction.

### Inferred Migration History

**Figure 4:**
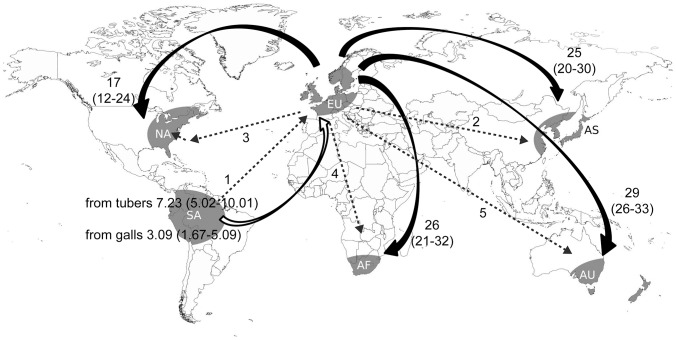
Global gene flow for *Spongospora subterranea* f.sp. *subterranea* and historic dissemination of the potato (*Solanum tuberosum*). Estimates were determined among Europe (EU), Africa (AF), Asia (AS), Australasia (AU), North America (NA), and South America (SA). The white arrow indicates estimates of historic gene flow using MIGRATE given as migrants scaled by the mutation rate. Curved black arrows indicate significant recent gene flow between regions inferred by BAYESASS, given as percent of the receiving population. Confidence intervals (5%–95%) are given in parenthesis. Numbered straight arrows represent the historic dissemination steps of potato mediated by human activities; 1) The conquistadors brought the potato to Europe (1567–1593); 2). In the early 1600s, the potato was taken from Europe to Asia; 3) In 1613, the potato was taken from England to Bermuda and from there to Virginia (United States) in 1620; 4 and 5) Further dissemination of potato from England to Southern Africa (1880s), New Zealand (1769) and Australia (1787). Data extracted from historic documents, provided by International Potato Center (CIP), Peru.

Historical migration rates *M* (scaled by the mutation rate) were estimated with MIGRATE-N. We found strong asymmetrical gene flow (Fig. 4). All estimates for South America tuber lesions and South America galls into Europe or into the pooled introduced regions had confidence ranges >1 and ranged from *M* = 2.14 to *M* = 7.23. In contrast, migration estimates for the opposite directions indicated no significant historical gene flow from Europe or the pooled introduced regions into South America with estimates ranging from 0.00 to 0.34 (Tables S5a, b).

We estimated recent migration rates between all regions using BayesAss. The most striking finding was the high proportion of European migrants in all other introduced populations, ranging from 17% in North America to 29% in Australasia (Fig. 4). In sharp contrast, the proportions of migrants originating from other regions than Europe were all non-significant from zero based on confidence intervals. In contrast to our estimates of past migration rates, we did not detect any significant recent gene flow between the South American regions and the introduced regions (Table S6).

## Discussion

The present study is the first detailed population genetic characterization of a plasmodiophorid plant pathogen. The determination of the genetic structure of *Sss* was challenging, because suitable genetic markers were lacking and plasmodiophorids are obligate biotrophic pathogens that cannot be grown as pure culture on artificial media [7]; [23]. For this reason, sequencing of housekeeping genes and the *ITS* region has a longer tradition in *Sss* than for other genotyping approaches [26]; [50]; [28]. We successfully genotyped 693 individual samples of *Sss* with six newly developed polymorphic microsatellite markers. The sporosori originated from all relevant continents, different climate zones, different potato subspecies and cultivars, and also from tuber lesions and root galls.

Our microsatellite and sequencing data showed that South American populations were consistently more diverse compared to all other regions. Estimates of migration rates further suggested a historic gene flow from South America to Europe and recent gene flow from Europe to the other introduced regions. Consequently, we conclude that *Sss* is very likely to have been introduced from South America to Europe and, with Europe as a “bridgehead”, was then further disseminated around the world, with no or a very limited number of new introductions from the native region.

### Role of Hosts, Inoculum and the Potential of Cryptic Taxa

Although our collection included *Sss* samples extracted from the three most common cultivated potato hosts *S. tuberosum* ssp. *tuberosum*, *S. tuberosum* ssp. *andigena*, and *S. phureja*, we found no evidence of genetic differences in *Sss* related to different hosts in either the sequence-based phylogeny reconstruction or in the microsatellite based cluster analysis. On Colombian potatoes, tuber lesion formation is rare [22]; for that reason, tuber lesion samples have been obtained from only one region in Colombia. However, we do not think that this sampling introduced a bias caused, for example, by regional adaptation, since *F_ST_* results showed that the Colombian tuber lesion samples clustered with those from the Venezuelan tuber lesions. On *S. phureja* only root galls can be found. This *Solanum* species may have specific resistance to tuber infection. Among the many cultivars of *S. tuberosum* ssp. *tuberosum*, which is the potato host widely grown in the introduced regions, both disease symptoms (root galls and tuber lesions) can be observed. However, there are also cultivar differences in susceptibility to disease on tubers and roots [21].

South American samples collected from galls were genetically distinct from those collected from lesions. No such distinction could be detected among gall and lesion samples from the introduced regions. This could indicate an ecological adaptation in the native regions due to co-evolutionary processes and/or competitive exclusion. This might be especially true in Colombia, where little or no potato exchange has occurred to date with other countries (E. Gilchrist, Corporación Universitaria Lasallista, Colombia, personal communication), and only S. *tuberosum* ssp *andigena* and *S. phureja* hosts are cropped. Further investigations should be conducted on samples from Colombia that could identifiy of different ecotypes or subspecies of *Sss*.

### Invasion Scenario of Sss and Evidence for a Bridgehead Effect

Given the very restricted dispersal ability of *Sss* and our samples covering six continents, we expected to find considerable regional substructure due to restricted gene flow, founder effects or local adaptations. However, our PCA analyses indicated shallow population substructures, both for the introduced and native regions. In contrast, we observed marked differences in genetic diversity between South America and the other regions. South American samples were genetically more variable, consistent with the hypothesis that the native region of *Sss* is South America – which is also the native region of potato. Reduced genetic diversity and lack of substructure in collections from introduced regions can be attributed to founder effects, and are indicative of a rapid global invasion process and/or restricted origin of all introduced populations, combined with a lack of recurrent gene flow from the native region.

The results of our migration analyses supported the hypothesis of a historic gene flow from South America into Europe. Furthermore, estimates of recent gene flow suggested no gene flow between South America and Europe, but that all introduced regions received migrants from Europe. We included historic documentation of dissemination of potato [30] to reconstruct the invasion scenario of *Sss*. In combination with our results, we hypothesize the following “bridgehead” invasion scenario of *Sss*. The native region and source of *Sss* is located in South America. It is most likely that *Sss* was introduced from South America to Europe on contaminated potato specimens, in the second half of the sixteenth century. This could possibly be through the exploration and migratory activities associated with the conquistador era. Supporting this is the first documented report of powdery scab published in Germany in 1842, describing the disease as a well-known problem for farmers [51]. From the bridgehead in Europe, *Sss* was spread subsequently due to the lack of plant quarantine or control measures to the North American and European colonies in Africa, Asia and Australasia. North America had the lowest genetic diversity of all introduced regions. Since potatoes were first introduced from Europe to the Bermudas and from there to Virginia, this possibly resulted in a secondary genetic bottleneck in the North American populations of *Sss*. No significant further exchange of infected potatoes has occurred between South America and the introduced regions since the first introduction to Europe.

To pinpoint the exact location of origin of *Sss*, a more thorough sampling of South American *Sss* populations is necessary. Results may reveal the region of origin of *Sss* to be in Peru, if the pathogen has co-evolved with the edible potato, by far the most important host today. This plant was first collected by hunter gatherers in the Lake Titicaca region in Peru around 3,000 B.C., and was later distributed to other Andean countries and cultivated in the first agricultural societies in South America [30]. Supporting this hypothesis is that the most basal haplotype (cHap19) of the *Sss* clade reconstructed in the phylogenetic tree was collected in Peru.

### Global Trade of Seed Potatoes Reflects Recent Migration Patterns

*Sss* is a soilborne obligate biotroph with very restricted natural dispersal abilities. The most likely way that *Sss* has been dispersed throughout the world is, therefore, via the movement and trade of seed potatoes. *Sss* can be transmitted on seed tubers, either as sporosori in visible powdery scab lesions or as non-visible surface contaminants [52]. In this way, the pathogen might invade new regions as a contaminating organism in consignments of shipped seed potatoes.

According to the recently published potato trade map [31], Europe, mainly the Netherlands, is by far the greatest exporter of seed potatoes worldwide. In accordance with our results, this strongly suggests that Europe is the contemporary global distributor of potatoes potentially infected with *Sss* to other introduced regions of the world.

### Risk Assessment for Sss, and Comparison with P. infestans

It can be assumed that several important potato pathogens were carried out of South America. This probably happened together with the potato but also possibly in contaminated soil or infected plant material. Parallels between *Sss* and other important potato pathogens can be found. Potato late blight is now considered a re-emerging disease. Several *P. infestans* introduction events took place in the nineteenth and twentieth centuries outgoing from Latin America to Europe. The pathogen reproduces sexually given the presence of the compatible mating types A1 and A2, but sexual reproduction was not observed until the mid-1970s, when potatoes were imported on large scale after a drought in 1976 [6]. Before this specific event, *P. infestans* reproduced asexually, and studies revealed that it was a worldwide clone [12]. A similar situation exists with other successful globally distributed and economically important potato pathogens, including the two bacterial wilt pathogens *Burkholderia solanacearum* and *Ralstonia solanacearum* [16]–[17]. Like *P. infestans* and the bacterial wilt pathogens, *Sss* was able to successfully invade regions far beyond its native range in South America despite its reduced genetic diversity. Multiple introductions may greatly modify the population genetic structure of plant pathogens in their new areas and influence their evolutionary potential [53]; [54]. We found a smaller proportion of clones in native South American samples compared to the introduced regions. However, estimates of I_A_^S^ for both introduced and native regions suggested a predominantly asexual reproduction. This is counter-intuitive, because the DNA was extracted from sporosori, a resting structure assumed to be the product of sexual recombination [25]. This knowledge gap on the occurrence of sexual reproduction in the life cycle of *Sss* remains to be adequately elucidated.

### Strategies for Controlling Sss and Potato Breeding

Chemical control measures for powdery scab are not completely reliable and are increasingly undesirable for environmental and consumer resistance reasons. The present study confirms that development of potato breeding lines and cultivars resistant to powdery scab is likely to be an efficient and sustainable way to manage the disease [23]. For example, high gene flow and regular sexual recombination were identified as important factors that increase the evolutionary risk of plant pathogens [53]. Gene flow allows the rapid distribution of virulent mutants across large geographical areas. Recombination allows a pathogen to put together new combinations of mutant alleles, and thus allows it to overcome the combination of major resistance genes deployed in crop breeding. *Sss* is a soil-borne pathogen and natural gene flow is therefore very low. Furthermore, given the great clonality of *Sss* in the introduced regions, resistance screening during breeding is not likely to be faced with variable virulence in pathogen populations due to recombination. The risk of virulence differences within the clonal lineages [55] seems to be low, as screening trials during 4 years with cultivars selected for their susceptibility to powdery scab showed no differences in the performance of the genotypes [56]. The cultivar ‘Gladiator’, bred in New Zealand [57] with very low *Sss* susceptibility to tuber and root infection, performed well in all years and locations, even in those where inoculum levels were high and severe powdery scab epidemics occurred.

The similarities between *Sss* and other potato pathogens must be considered. New introductions of *Sss* genotypes, particularly from South America, would increase the potential of more aggressive inoculum, e.g. due to recombination. This could lead to multiple pathotypes and additional challenges for resistance breeding. In order to prevent such introductions, strict quarantine measures for potato import need to be established, or where they exist, strictly enforced. This will help to preserve the long-term benefit of resistant cultivars and maintain low genetic variability of the pathogen on a global scale.

## Supporting Information

Figure S1 **Concatenated ITS and** ***actin*** **haplotype network inferred by the software TCS from sequencing data of 308 global samples of** ***Spongospora subterranea*** **f.sp.** ***subterranea*** **.**
(PDF)

Table S1 ***Spongospora subterranea*** **f.sp.** ***subterranea*** **samples examined.**
(DOC)

Table S2 **PCR protocols and primers used to amplify microsatellite loci and partial sequences of the** ***actin*** **gene and** ***ITS*** **region in** ***Spongospora subterranea*** **f.sp.** ***subterranea*** **.** Numbers of repeated microsatellite (Msat) motifs refer to the *Sss* sample used for primer designs.
(DOC)

Table S3 **Absolute numbers and allele frequencies (in brackets) for all 693 samples, genotyped with six loci (Supplementary** **Table 2** **), estimated by GENODIVE.** The top numbers indicate the allele length in base-pairs. The total number of alleles detected at all loci was 35.
(DOC)

Table S4 **Distribution of** ***Spongospora subterranea*** **f.sp.** ***subterranea ITS*** **and** ***actin*** **haplotypes.** The haplotypes occurring in each global region are listed for both sequences. This is visualized in Fig. S1.
(DOC)

Table S5 **(a) MIGRATE estimates of past migration rates** ***M*** **for** ***Spongospora subterranea*** **f.sp.** ***subterranea*** **between Europe and introduced regions. 0.05 and 0.95 percentiles indicated in parentheses.** **(b)** MIGRATE estimates of past migration rates *M* for *Spongospora subterranea* f.sp. *subterranea* between native and pooled introduced regions. 0.05 and 0.95 percentiles indicated in parentheses.
(DOC)

Table S6 **Estimates of recent migration rates (% of immigrant origin) of** ***Spongospora subterranea*** **f.sp.** ***subterranea*** **between native and introduced regions.** 95% confidence intervals indicated in parentheses.
(DOC)
