# Supplementary figures and images for: Global Genetics and Invasion History of the Potato Powdery Scab Pathogen, Spongospora subterranea f.sp. subterranea
Source: PLoS One. 2013 Jun 28;8(6):e67944. doi: 10.1371/journal.pone.0067944 (PMC3695870; doi:10.1371/journal.pone.0067944)

# Concatenated haplotype network of *ITS* and *actin* sequences

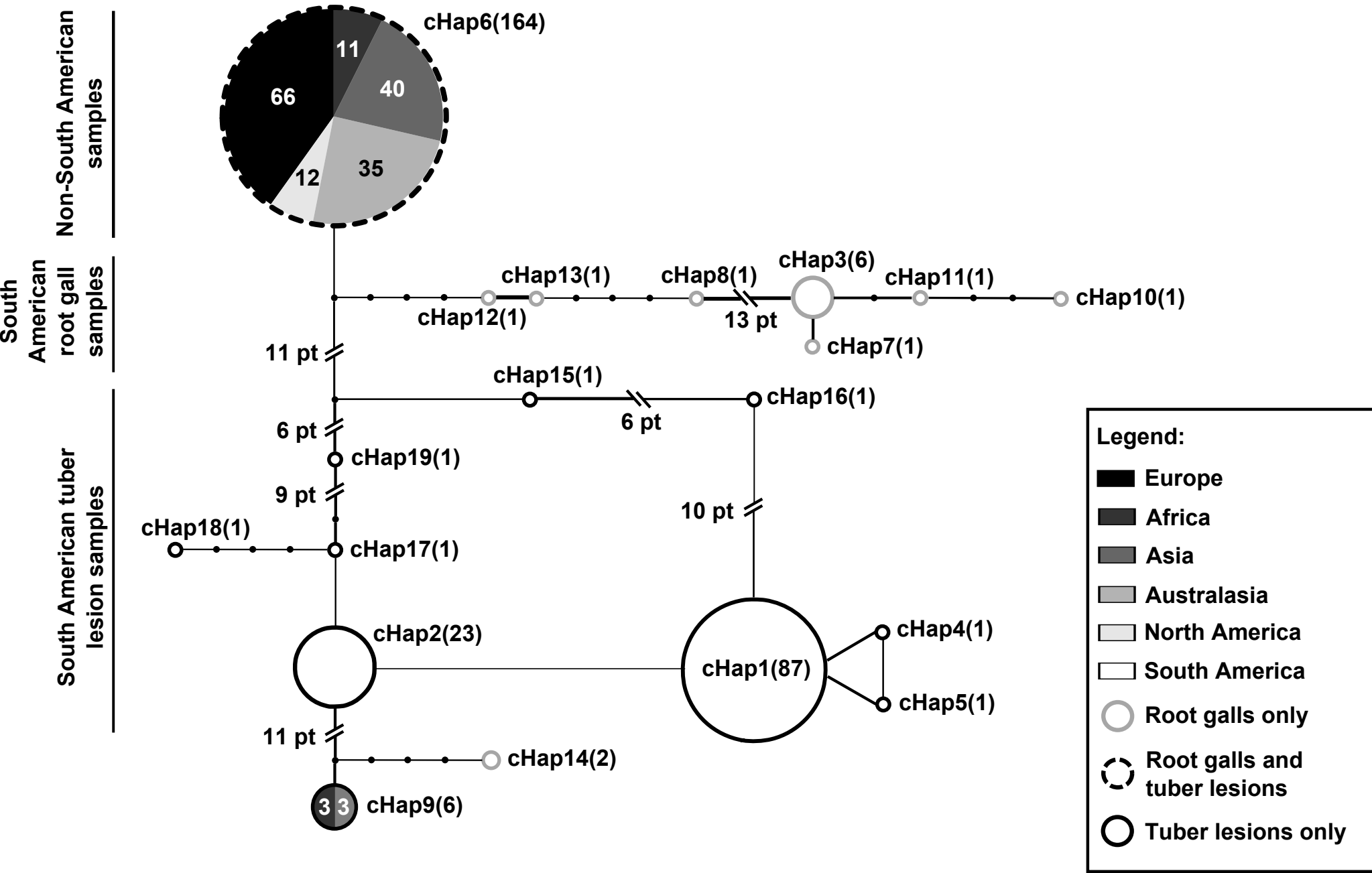

Supplement: Figure S1 — Concatenated ITS and actin haplotype network inferred by the software TCS from sequencing data of 308 global samples of Spongospora subterranea f.sp. subterranea . (PDF) [file pone.0067944.s001.pdf]
