# Supplementary material for: Global Genetics and Invasion History of the Potato Powdery Scab Pathogen, Spongospora subterranea f.sp. subterranea
Source: PLoS One. 2013 Jun 28;8(6):e67944. doi: 10.1371/journal.pone.0067944 (PMC3695870; doi:10.1371/journal.pone.0067944)
Supplement: Table S1 — Spongospora subterranea f.sp. subterranea samples examined. (DOC) [file pone.0067944.s002.doc]

**Supplementary Table 1** *Spongospora subterranea* f.sp. *subterranea* samples examined.

| Origin | Number of sites | Number of samples (N) | Solanum subspecies | Cultivar | Tissue | Year |
| --- | --- | --- | --- | --- | --- | --- |
| Switzerland, Europe | | | | | | |
| Wallestalden, Langnau | 1 | 31 | *Tuberosum* | Antonia, Talent, Rosagold, Pepite, BP, GO2TT118004, Bintje, Agria, Zorba, Mirage, AR98-1196, AR99-1200, ST98-74-9, G00SC235, Erika, GO2TT118004, B00/244/51, VR-98-72, Salome, Musica, Challenge | Lesions | 2009 |
| Wallestalden, Langnau | 2 | 30 | *Tuberosum* | Estima, Ratte, Saturna | Lesions and Galls | 2004, 2005 |
| Solothurn, Biezwil | 1 | 25 | *Tuberosum* | Agria | Lesions | 2004 |
| Gebr. Kobel | 1 | 1 | *Tuberosum* | Agria | Lesions | 1998 |
| Semag | 7 | 7 | *Tuberosum* | Agria, Erntestolz, Markies, Bintje | Lesions | 1998, 2000, 2003, |
| Luzern, Kägiswil | 2 | 19 | *Tuberosum* | Agria | Lesions | 2004 |
| *Germany, Europe* | | | | | | |
| Nordrhein-Westfalen, Meinersen | 1 | 23 | *Tuberosum* | Agria, E 05/421/529, M 05/127/29, E 05/60/8, E 05/251/215, E 05/99/45, B 05/251/115, B 05/139/34, M 05/68/34, SP 05/582/58, Seedling in peat soil | Lesions and Galls | 2009 |
| *Netherlands, Europe* | | | | | | |
| Limburg | 1 | 3 | *Tuberosum* | Unknown | Lesions | 2009 |
| Unknown | 1 | 1 | *Tuberosum* | Bintje | Lesions | 2000 |
| *Norway, Europe* | | | | | | |
| Farmen, Kvelde | 1 | 25 | *Tuberosum* | Redstar | Lesions | 2008 |
| Rustad, Romeldal | 1 | 25 | *Tuberosum* | Beate | Lesions | 2008 |
| *Iceland, Europe* | | | | | | |
| South Iceland, Thykkvibær | 1 | 22 | *Tuberosum* | Red Icelandic | Lesions | 2009 |
| *Scotland, Europe* | | | | | | |
| Scotland, Aberdeen | 3 | 3 | *Tuberosum* | Estima, Nadine, Mixture | Lesions | 1999 |
| *Sweden, Europe* | | | | | | |
| Uppsala | 2 | 2 | *Tuberosum* | Kultivator | Lesion scrapings | 1994, 2004 |
| *South Africa, Africa* | | | | | | |
| Kwazulu-Natal & Sandvelt | 1 | 25 | *Tuberosum* | Mondial | Lesions | 2009 |
| Sandvelt, Western Cape | 1 | 32 | *Tuberosum* | Mondial | Lesions | 2009 |
| *Pakistan, Asia* | | | | | | |
| Sharan, Kaghan Valley | 1 | 16 | *Tuberosum* | Cardinal, Barma | Lesions | 1994, 2009 |
| *Sri Lanka, Asia* | | | | | | |
| Unknown | 1 | 4 | *Tuberosum* | Mixture, Calwhite, Redlasoda, Chieftain, Keuka Gold, Granola | Galls | 2010 |
| *South Korea, Asia* | | | | | | |
| Hoenggye | 1 | 25 | *Tuberosum* | Superior | Lesions | 2010 |
| Wangsan | 1 | 25 | *Tuberosum* | Superior | Lesions | 2010 |
| Unknown | 1 | 1 | *Tuberosum* | Unknown, minitubers | Lesions | 2005 |
| *Japan, Asia* | | | | | | |
| Hokkaido, Kyogoku-Town | 1 | 25 | *Tuberosum* | Irish cobbler | Lesions | 2009 |
| Hokkaido, Unknown | 1 | 3 | *Tuberosum* | Unknown (KNP, QNO, TUM) | Lesions | 1999 |
| *Australia, Australasia* | | | | | | |
| Ballarat | 3 | 57 | *Tuberosum* | Unknown | Lesions | 2006 |
| *New Zealand, Australasia* | | | | | | |
| New Zealand, Canterbury, Lincoln | 1 | 106 | *Tuberosum* | Rua, Agria, and Desiree | Lesions | 1997, 2004, 2008 |
| New Zealand, Canterbury, Lincoln | 1 | 1 | *Solanum chacosense* | Bolivian Weed | Lesions | 2008 |
| New Zealand, Canterbury, Lincoln | 1 | 3 | *Tuberosum* | Shepody, Kennebec, Iwa | Lesions | 2008 |
| New Zealand, Canterbury, Lincoln | 1 | 1 | *Tuberosum* | Ranger Russet | Galls | 2008 |
| New Zealand, Canterbury, Lincoln | 1 | 2 | *S. phylum* | Unknown | Galls | 2008 |
| *United States of America, North America* | | | | | | |
| Pennsylvania, Potter County | 1 | 25 | *Tuberosum* | Shepody, Yukon Gold, B1992/106, AF2376-5, NY139, BC001357-4, FL30, W2310-3, Dakota Jewel, W2133-1, Atlantic, NYB38-40, Katahdin, Snowden, Dakota Diamond, Superior, Kennebec, Beacon Chipper, Dark Red Norland, Chieftain, NY141, BC001306-2, FL22, AF2393-7, B2452-3 | Lesions | 2009 |
| Madras, Oregon | 1 | 1 | *Tuberosum* | NDO 1496-1 | Lesions | 1995 |
| *Colombia, South America* | | | | | | |
| Villapinzón, Sonsa Bajo | 1 | 18 | *Andigena* or *Phureja* | Unknown | Lesions | 2008 |
| Nariño, Coba Negra | 1 | 4 | *Andigena* or *Phureja* | Unknown | Galls | 2008 |
| Nariño, La Marqueza | 1 | 5 | *Andigena* or *Phureja* | Unknown | Galls | 2008 |
| Nariño, Río Bobo | 1 | 6 | *Andigena* or *Phureja* | Unknown | Galls | 2008 |
| La Union, Antioquia | 1 | 25 | *Phureja* | Chuscalito, Buena Vista, La Madera Soka, Valluelitos Zuia la Cana, Valluelito Capiro, Don Juan de Jesu, Chuscalito Capiro | Galls | 2010 |
| *Venezuela, South America* | | | | | | |
| Mérida State, El Llano | 1 | 24 | *Andigena* | No Name | Lesions | 2010 |
| Mérida State, Los Muros de Tadeo | 1 | 25 | *Andigena* | Unica | Lesions | 2010 |
| Mérida State, La Toma | 1 | 22 | *Andigena* | Unica | Lesions | 2010 |
| Mérida State | 3 | 80 | *Tuberosum* | Granola | Lesions and 1 gall sample | 2011 |
| *Peru, South America* | | | | | | |
| Peru | 1 | 1 | *Andigena* | Unknown | Lesions | 1996 |
| Peru | 1 | 1 | *Tuberosum-Andigena* Hybrid | Mariva | Lesions | 1999 |
| *Ecuador, South America* | | | | | | |
| Ecuador | 1 | 1 | *Andigena* | Unknown | Lesions | 2000 |
