# Supplementary material for: Global Genetics and Invasion History of the Potato Powdery Scab Pathogen, Spongospora subterranea f.sp. subterranea
Source: PLoS One. 2013 Jun 28;8(6):e67944. doi: 10.1371/journal.pone.0067944 (PMC3695870; doi:10.1371/journal.pone.0067944)
Supplement: Table S2 — PCR protocols and primers used to amplify microsatellite loci and partial sequences of the actin gene and ITS region in Spongospora subterranea f.sp. subterranea . Numbers of repeated microsatellite (Msat) motifs refer to the Sss sample used for primer designs. (DOC) [file pone.0067944.s003.doc]

**Table S2** PCR protocols and primers used to amplify microsatellite loci and partial sequences of the *actin* gene and *ITS* region in *Spongospora subterranea* f.sp. *subterranea.* Numbers of repeated microsatellite (Msat) motifs refer to the *Sss* sample used for primer designs.

| Marker Name | Synthetic Fragment Length [bp] | Motif | Label | Primer Sequence from 5’ |
| --- | --- | --- | --- | --- |
| Act | ~650 | *Actin* gene outer region | none | F: ACTCCGGCCATGTATGTCG R: TGCCTCGTCATACTCATCTTTG |
| ActN | 615 | *Actin* gene inner region | none | F: GGCTGTTCTATCTCTATACGCATC R: CGACAACGATGACAAAATCG |
| Msat6 | 210 | (GAC/CAC)_8_ | PET | F: PET-GGGAGATCAGCTCCAGATCA R: GGTGTCGTTGTTAGGGTTGC |
| Msat45 | 268 | (TCA)_7_ | NED | F: NED-GAGCGAAACTGAGGGGATTC R: CCTTCTCCAGGATCGGGATC |
| Msat84 | 291 | (GCT)_6_ | VIC | F: VIC-GAGCTAGACCTACCGACGACT R: ACGTCAGTGATCCAAGCACA |
| Msat103 | 193 | (CT)_12_ | HEX | F: HEX-GATGATCGTCGGAATTCGTT R: CATCTCGAGCTTCGTTCAGC |
| Msat246.1 | 140 | (CAA)_6_ | FAM | F: FAM-CCAGACAACCCCTGTTCAGT R:CCAAGCGTTAACCCACTGTT |
| Msat246.2 | 160 | (CAA)_5_ | FAM | F: FAM-CCAGACAACCCCTGTTCAGT R:CCAAGCGTTAACCCACTGTT |
|  |  |  |  |  |
| Protocol *Actin* PCRs | 94°C for 3 min initial denaturation; 45 cycles of 92°C for 45 s, 50°C for 1 min, 72°C for 1:30 min; and 72°C for 7 min final elongation | | | |
| Mix *Actin* PCRs: | To obtain *actin* amplicons, nested PCRs consisting of two sequential PCR steps were performed. First with an outer and second with an inner primer pair. For the first step, 5 µl of genomic DNA (15 to 20 ng of final concentration) were added to 20 µl volumes of 2 µl 10 x DreamTAQ PCR buffer including 25 mM of MgCl2, 1 µl 2.5 mM dNTP, 0.5 µl 5 mM forward primer Act_F, 0.5 µl 5 mM reverse primer Act_R and 1 unit DreamTAQ polymerase (Fermentas). The PCR protocol started with an initial denaturation for 3 min at 94°C, followed by 45 cycles of denaturation for 45 s at 92°C, annealing for 50 s at 50°C and elongation for 1:30 min at 72°C, and the protocol ended with a final extension step for 5 min at 72°C. For the second step, the products resulting from the first step were diluted 1:20 and 3 µl of the dilution were added to 20 µl volumes of 2 µl 10 x DreamTAQ PCR buffer including 25 mM of MgCl_2_, 1 µl 2.5 mM dNTP, 0.5 µl 5 mM forward primer ActN_F, 0.5 µl 5 mM reverse primer ActN_R and 1 unit DreamTAQ polymerase (Fermentas). The PCR protocol was the same as in the first step. | | | |
| Protocol and Mix for *ITS* PCRs | As in Bulman & Marshall (1998) | | | |
| Protocol Microsatellite PCRs | 95°C for 2:30 min initial denaturation; 35 cycles of 95°C for 40 s, 55°C for 30 s, 72°C for 30 s; and 72°C for 7 min final elongation | | | |
| Mix Microsatellite PCRs | 20 µl volumes containing 5 µl of genomic DNA (15 to 20 ng final concentration), 2 µl 10 x DreamTAQ PCR buffer (Fermentas) including 25 mM MgCl_2_, 0.2 mM dNTP, 20 nM labeled forward primer, 90 nM unlabeled forward primer, 100 nM unlabeled reverse primer and 1 unit DreamTAQ polymerase (Fermentas) | | | |
