# Supplementary material for: Global Genetics and Invasion History of the Potato Powdery Scab Pathogen, Spongospora subterranea f.sp. subterranea
Source: PLoS One. 2013 Jun 28;8(6):e67944. doi: 10.1371/journal.pone.0067944 (PMC3695870; doi:10.1371/journal.pone.0067944)
Supplement: Table S3 — Absolute numbers and allele frequencies (in brackets) for all 693 samples, genotyped with six loci (Supplementary Table 2 ), estimated by GENODIVE. The top numbers indicate the allele length in base-pairs. The total number of alleles detected at all loci was 35. (DOC) [file pone.0067944.s004.doc]

**Table S3** Absolute numbers and allele frequencies (in brackets) for all 693 samples, genotyped with six loci (Supplementary Table 2), estimated by GENODIVE. The top numbers indicate the allele length in base-pairs. The total number of alleles detected at all loci was 35.

| Msat6_GAC/CAC | | | | | | | | |  |
| --- | --- | --- | --- | --- | --- | --- | --- | --- | --- |
| Population | Total | Missing | 204 | 207 | 210 | 225 | 231 | 237 | |
| Europe | 430 | 0 | 0 | 430 (1) | 0 | 0 | 0 | 0 | |
| Africa | 114 | 0 | 0 | 114 (1) | 0 | 0 | 0 | 0 | |
| Asia | 196 | 0 | 0 | 192 (0.98) | 2 (0.01) | 0 | 2 (0.01) | 0 | |
| Australasia | 340 | 0 | 0 | 340 (1) | 0 | 0 | 0 | 0 | |
| North America | 52 | 0 | 0 | 52 (1) | 0 | 0 | 0 | 0 | |
| South American root galls | 76 | 0 | 2 (0.026) | 3 (0.039) | 3 (0.039) | 1 (0.013) | 67 (0.882) | 0 | |
| South American tuber lesions | 178 | 0 | 0 | 14 (0.079) | 158 (0.888) | 0 | 5 (0.028) | 1 (0.006) | |
| Overall | 1386 | 0 | 2 (0.001) | 1145 (0.826) | 163 (0.118) | 1 (0.001) | 74 (0.053) | 1 (0.001) | |

| Msat45_TCA | | | | | | |
| --- | --- | --- | --- | --- | --- | --- |
| Population | Total | Missing | 265 | 268 | 271 | 274 |
| Europe | 430 | 0 | 12 (0.028) | 418 (0.972) | 0 | 0 |
| Africa | 114 | 0 | 0 | 114 (1) | 0 | 0 |
| Asia | 196 | 0 | 0 | 191 (0.974) | 1 (0.005) | 4 (0.02) |
| Australasia | 340 | 0 | 0 | 340 (1) | 0 | 0 |
| North America | 52 | 0 | 0 | 52 (1) | 0 | 0 |
| South American root galls | 76 | 0 | 0 | 14 (0.184) | 20 (0.263) | 42 (0.553) |
| South American tuber lesions | 178 | 0 | 0 | 1 (0.006) | 1 (0.006) | 176 (0.989) |
| Overall | 1386 | 0 | 12 (0.009) | 1130 (0.815) | 22 (0.016) | 222 (0.16) |

| Msat84_GCT | | | | | | | | | | | | | | |
| --- | --- | --- | --- | --- | --- | --- | --- | --- | --- | --- | --- | --- | --- | --- |
| Population | Total | Missing | 258 | 261 | 270 | 274 | 279 | 282 | 288 | 290 | 291 | 294 | 297 | 303 |
| Europe | 430 | 0 | 0 | 0 | 0 | 1 (0.002) | 0 | 0 | 0 | 0 | 426 (0.991)) | 3 (0.007) | 0 | 0 |
| Africa | 114 | 0 | 6 (0.053) | 2 (0.018) | 1 (0.009) | 0 | 1 (0.009) | 0 | 2 (0.018) | 1 (0.009) | 99 (0.868) | 0 | 2 (0.018) | 0 |
| Asia | 196 | 0 | 0 | 0 | 0 | 0 | 0 | 0 | 0 | 0 | 196 (1) | 0 | 0 | 0 |
| Australasia | 340 | 0 | 0 | 0 | 0 | 0 | 0 | 0 | 0 | 0 | 340 (1) | 0 | 0 | 0 |
| North America | 52 | 0 | 0 | 0 | 0 | 0 | 0 | 0 | 0 | 0 | 52 (1) | 0 | 0 | 0 |
| South American  root galls | 76 | 0 | 0 | 0 | 0 | 0 | 0 | 0 | 0 | 0 | 5 (0.066) | 24 (0.316) | 0 | 47 (0.618) |
| South American  tuber lesions | 178 | 0 | 0 | 0 | 0 | 0 | 0 | 1 (0.006) | 0 | 0 | 0 | 167 (0.938) | 2 (0.011) | 8 (0.045) |
| Overall | 1386 | 0 | 6 (0.004) | 2 (0.001) | 1 (0.001) | 1 (0.001) | 1 (0.001) | 1 (0.001) | 2 (0.001) | 1 (0.001) | 1118 (0.807) | 194 (0.14) | 4 (0.003) | 55 (0.04) |

| Msat103_CT | | | | | | | | | |
| --- | --- | --- | --- | --- | --- | --- | --- | --- | --- |
| Population | Total | Missing | 179 | 187 | 191 | 193 | 195 | 197 | 199 |
| Europe | 430 | 0 | 0 | 0 | 0 | 136 (0.316) | 0 | 292 (0.679) | 2 (0.005) |
| Africa | 114 | 0 | 2 (0.018) | 0 | 0 | 69 (0.605) | 0 | 43 (0.377) | 0 |
| Asia | 196 | 0 | 0 | 1 (0.005) | 0 | 127 (0.648) | 2 (0.01) | 64 (0.327) | 2 (0.01) |
| Australasia | 340 | 0 | 0 | 0 | 0 | 249 (0.732) | 1 (0.003) | 89 (0.262) | 1 (0.003) |
| North America | 52 | 0 | 0 | 0 | 0 | 1 (0.019) | 0 | 50 (0.962) | 1 (0.019) |
| South American root galls | 76 | 0 | 0 | 0 | 0 | 2 (0.026) | 0 | 3 (0.039) | 71 (0.934) |
| South American tuber lesions | 178 | 0 | 0 | 0 | 8 (0.045) | 99 (0.556) | 0 | 51 (0.287) | 20 (0.112) |
| Overall | 1386 | 0 | 2 (0.001) | 1 (0.001) | 8 (0.006) | 683 (0.493) | 3 (0.002) | 592 (0.427) | 97 (0.07) |

| Msat246.1_CAA | | | | |
| --- | --- | --- | --- | --- |
| Population | Total | Missing | 140 | 142 |
| Europe | 430 | 0 | 112 (0.26) | 318 (0.74) |
| Africa | 114 | 0 | 23 (0.202) | 91 (0.798) |
| Asia | 196 | 0 | 63 (0.321) | 133 (0.679) |
| Australasia | 340 | 0 | 88 (0.259) | 252 (0.741) |
| North America | 52 | 0 | 0 | 52 (1) |
| South American root galls | 76 | 0 | 27 (0.355) | 49 (0.645) |
| South American tuber lesions | 178 | 0 | 54 (0.303) | 124 (0.679) |
| Overall | 1386 | 0 | 367 (0.265) | 1019 (0.735) |

| Msat246.2_CAA | | | | | | |
| --- | --- | --- | --- | --- | --- | --- |
| Population | Total | Missing | 156 | 158 | 160 | 168 |
| Europe | 430 | 0 | 147 (0.324) | 282 (0.656) | 1 (0.002) | 0 |
| Africa | 114 | 0 | 25 (0.219) | 89 (0.781) | 0 | 0 |
| Asia | 196 | 0 | 85 (0.434) | 111 (0.566) | 0 | 0 |
| Australasia | 340 | 0 | 136 (0.4) | 204 (0.6) | 0 | 0 |
| North America | 52 | 0 | 0 | 52 (1) | 0 | 0 |
| South American root galls | 76 | 0 | 56 (0.737) | 20 (0.263) | 0 | 0 |
| South American tuber lesions | 178 | 0 | 91 (0.511) | 85 (0.478) | 0 | 2 (0.011) |
| Overall | 1386 | 0 | 540 (0.39) | 843 (0.608) | 1 (0.001) | 2 (0.001 |
