# Supplementary material for: Global Genetics and Invasion History of the Potato Powdery Scab Pathogen, Spongospora subterranea f.sp. subterranea
Source: PLoS One. 2013 Jun 28;8(6):e67944. doi: 10.1371/journal.pone.0067944 (PMC3695870; doi:10.1371/journal.pone.0067944)
Supplement: Table S4 — Distribution of Spongospora subterranea f.sp. subterranea ITS and actin haplotypes. The haplotypes occurring in each global region are listed for both sequences. This is visualized in Fig. S1. (DOC) [file pone.0067944.s005.doc]

**Table S4** Distribution of *Spongospora subterranea* f.sp. *subterranea* *ITS* and *actin* haplotypes. The haplotypes occurring in each global region are listed for both sequences. This is visualized in Fig. S1.

| Region | *ITS* Haplotypes (iHap) | *Actin* Haplotypes (aHap) | Concatenated Haplotypes (cHap) |
| --- | --- | --- | --- |
| Europe | 6 | 1, 3 | 6, 9 |
| Africa | 6 | 3 | 6 |
| Asia | 6 | 1, 3 | 6, 9 |
| Australasia | 6 | 3 | 6 |
| North America | 6 | 3 | 6 |
| Root galls South America | 3 | 1, 2, 3, 4, 5, 6, 7, 8 | 3, 7, 8, 10, 11, 12, 13, 14 |
| Tuber lesions South America | 1, 2, 4, 5, 7 | 1, 3, 9, 10, 11, 12 | 1, 2, 4, 5, 15, 16, 17, 18, 19 |
| Total South America | 1, 2, 3, 4, 5, 7 | 1, 2, 3, 4, 5, 6, 7, 8, 9, 10, 11, 12 | 1, 2, 3, 4, 5, 7, 8, 10, 11, 12, 13, 14, 15, 16, 17, 18, 19 |
