# Supplementary material for: Global Genetics and Invasion History of the Potato Powdery Scab Pathogen, Spongospora subterranea f.sp. subterranea
Source: PLoS One. 2013 Jun 28;8(6):e67944. doi: 10.1371/journal.pone.0067944 (PMC3695870; doi:10.1371/journal.pone.0067944)
Supplement: Table S5 — (a) MIGRATE estimates of past migration rates M for Spongospora subterranea f.sp. subterranea between Europe and introduced regions. 0.05 and 0.95 percentiles indicated in parentheses. (b) MIGRATE estimates of past migration rates M for Spongospora subterranea f.sp. subterranea between native and pooled introduced regions. 0.05 and 0.95 percentiles indicated in parentheses. (DOC) [file pone.0067944.s006.doc]

**Table S5a** MIGRATE estimates of past migration rates *M* for *Spongospora subterranea* f.sp. *subterranea* between Europe and introduced regions. 0.05 and 0.95 percentiles indicated in parentheses.

|  | Recipient of migrants | | |
| --- | --- | --- | --- |
| Source of migrants | EU | SA lesions | SA galls |
| Europe | - | 0.00 (0.00 – 0.00) | 0.34 (0.29 – 0.61) |
| South America tuber lesions | 7.23 (5.02 – 10.01) | - | 9.68 (7.62 – 12.09) |
| South America root galls | 3.09 (1.67 – 5.09) | 9.27 (7.39 – 11.37) | - |

**Table S5b** MIGRATE estimates of past migration rates *M* for *Spongospora subterranea* f.sp. *subterranea* between native and pooled introduced regions. 0.05 and 0.95 percentiles indicated in parentheses.

|  | Recipient of migrants | | |
| --- | --- | --- | --- |
| Source of migrants | Introduced regions | SA lesions | SA galls |
| Introduced regions  pooled | - | 0.00 (0.00 – 0.18) | 0.13 (0.11 – 0.40) |
| South America tuber lesions | 2.14 (1.37 – 3.12) | - | 2.22 (1.37 – 3.34) |
| South America root galls | 5.07 (3.90 – 6.51) | 7.52 (5.94 – 9.38) | - |
