# Supplementary material for: Global Genetics and Invasion History of the Potato Powdery Scab Pathogen, Spongospora subterranea f.sp. subterranea
Source: PLoS One. 2013 Jun 28;8(6):e67944. doi: 10.1371/journal.pone.0067944 (PMC3695870; doi:10.1371/journal.pone.0067944)
Supplement: Table S6 — Estimates of recent migration rates (% of immigrant origin) of Spongospora subterranea f.sp. subterranea between native and introduced regions. 95% confidence intervals indicated in parentheses. (DOC) [file pone.0067944.s007.doc]

**Table S6** Estimates of recent migration rates (% of immigrant origin) of *Spongospora subterranea* f.sp. *subterranea* between native and introduced regions. 95 % confidence intervals indicated in parentheses.

|  | Recipient of migrants | | | | | | |
| --- | --- | --- | --- | --- | --- | --- | --- |
|  | Introduced regions | | | | | Native regions | |
| Source of migrants | EU | AF | AS | AU | NA | SA lesions | SA galls |
| Europe | 99 (98 – 100) | 26 (21 – 32) | 25 (20 - 30) | 29 (26 - 33) | 17 (12 - 24) | 0 (0 – 1) | 1 (0 – 3) |
| Africa | 0 (0 – 1) | 69 (67 - 75) | 1 (0 - 3) | 0 (0 - 2) | 2 (0 – 4) | 0 (0 – 1) | 0 (0 – 1) |
| Asia | 0 (0 – 1) | 1 (0 - 3) | 71 (67 - 77) | 1 (0 - 2) | 2 (0 – 4) | 0 (0 – 1) | 0 (0 – 1) |
| Australasia | 0 (0 – 1) | 1 (0 - 3) | 1 (0 - 3) | 69 (67 – 70) | 2 (0 – 4) | 0 (0 – 1) | 0 (0 – 1) |
| North America | 0 (0 – 1) | 1 (0 - 3) | 1 (0 - 2) | 0 (0 - 2) | 75 (67 – 84) | 0 (0 – 1) | 0 (0 – 1) |
| South America tuber lesions | 0 (0 – 1) | 0 (0 - 2) | 1 (0 - 3) | 1 (0 - 3) | 1 (0 - 3) | 99 (98 – 100) | 1 (0 – 3) |
| South America root galls | 0 (0 – 1) | 0 (0 - 2) | 1 (0 - 3) | 1 (0 - 3) | 1 (0 - 3) | 0 (0 – 1) | 97 (94 – 99) |
